# Surface-Grinding-Induced Recrystallization and Metal Flow Causes Corrosion-Assisted Penetrating Attack of High-Mn–Low-CR Casting Steel in Humid Environments

**DOI:** 10.3390/ma17235922

**Published:** 2024-12-03

**Authors:** Jin Sung Park, Myeong Hun Kang, Sung Jin Kim

**Affiliations:** 1 Department of Materials Science and Engineering, Sunchon National University, Suncheon 57922, Republic of Korea; 2 POSCO Technical Research Laboratories, Gwangyang 57807, Republic of Korea; kangmh@posco.com

**Keywords:** grinding, high-Mn steel casting slab, corrosion, metal streamline, humidity level

## Abstract

This study examined the surface-grinding-induced microstructural modifications and corrosion attacks in a penetrating form of a high-Mn–low-Cr casting steel slab under humid environments. Various experimental and analytical findings from field-emission scanning electron microscopy, electron backscatter diffraction, transmission electron microscopy, and electrochemical analyses revealed that the abrasive grinding process led to the formation of a surface deformed region, comprising a recrystallized fine grain layer and multiple streamlines. Corrosion initially occurs preferentially along the boundary areas where Cr(Mn)_23_C_6_ particles are precipitated. Moreover, the corrosion products (Fe-based oxy/hydroxides) with a high volumetric expansion ratio detach readily from the surface deformed regions, facilitating the easy penetration of corrosive media. In contrast to conventional low-alloyed steels, which exhibit uniform corrosion behavior, corrosion-assisted penetrating attacks on ground high-Mn–low-Cr casting steel slabs occur more severely and frequently during the summer/dry season (i.e., relative humidity levels around 60% to 80%, rather than 100%) when a thin water film can form on the steel surface. Based on the result, effective technical strategies in terms of metallurgical and environmental aspects to mitigate the risk of corrosion-assisted penetrating attack of high-Mn–low-Cr casting steel were discussed.

## 1. Introduction

High-Mn–low-Cr steel alloys with a face-centered cubic (FCC) structure at room temperature have attracted significant attention owing to their desirable combination of superior mechanical properties and long-term lifespan even in harsh chloride environments [1,2,3]. Their intermediate products (e.g., casting slab), however, can sometimes fail, caused mainly by various types of surface and subsurface damage during subsequent manufacturing processes. Among these, the high temperature and severe plastic deformation provided by the surface grinding process can significantly modify the physical, mechanical, and electrochemical properties [4,5,6]. This modification may involve phase transformation, crystallographic texture modifications, work hardening, thermal softening, and dynamic softening including recovery and recrystallization [4,7,8]. According to Yao et al. [9], when grinding Cr-bearing steel using a flat grinder, its outer surface can be exposed to high temperatures of more than 1000 °C, leading to phase transformations. Moreover, the sample surface undergoes severe plastic deformation due to shearing and extrusion by abrasive particles. This abrasive grinding process is often regarded as a superb machining strategy for structural steel alloys, achieving a high-quality mechanically strengthened layer owing to the mechanical–thermal coupling effect [9,10]. On the other hand, the microstructural modifications on the distorted surface generated by severe plastic deformation can increase electrochemical reactivity, leading to a higher susceptibility to anodic metal dissolution of the surface.

It is known that adding even small quantities of Cr to various steel alloys, including the high-Mn–low-Cr steel alloys investigated in this work, enhances corrosion resistance in aqueous media [11,12,13,14,15,16,17,18]. Specifically, alloying 3 wt.% Cr to 24 wt.% Mn-bearing steel alloys significantly improves corrosion resistance in brine environments by forming bi-layered corrosion products, consisting of an inner Cr-enriched oxide (or hydroxide) and an outer Fe(Mn)-based oxide (or hydroxide) [12,13]. However, the intermediate products of high-Mn–low-Cr steel, such as casting slabs, may have inferior corrosion resistance primarily due to the formation of coarse grains and (Cr,Mn)-bearing precipitates resulting from slow cooling rates during the casting process. Additionally, their corrosion behaviors, such as corrosion type and kinetics, can depend greatly on environmental conditions to which they are exposed, such as temperature and humidity levels. Despite the considerable body of literature analyzing the mechanical properties of steel alloys undergoing the abrasive grinding process [4,5,6,7,8,9,10], the corrosion behavior of ground high-Mn–low-Cr cast steels in humid environments has not been mechanistically investigated.

The primary objective of the present study is to elucidate the relationship between abrasive grinding-induced microstructural changes and corrosion behaviors of high-Mn–low-Cr cast steel under humid environments. To accomplish this, a range of microstructural characterizations via field-emission scanning electron microscopy (FE-SEM) (Hitachi, Tokyo, Japan), electron backscatter diffraction (EBSD) (Symmetry, Oxford, UK), and transmission electron microscopy (TEM) (SELMI, Sumy, Ukraine) were conducted. Additionally, corrosion-induced surface degradation in humid environments was examined through periodic water spraying and simulated temperature–humidity exposure testing, followed by surface analysis using FE-SEM.

## 2. Experimental

### 2.1. Material Preparation

**Figure 1:**
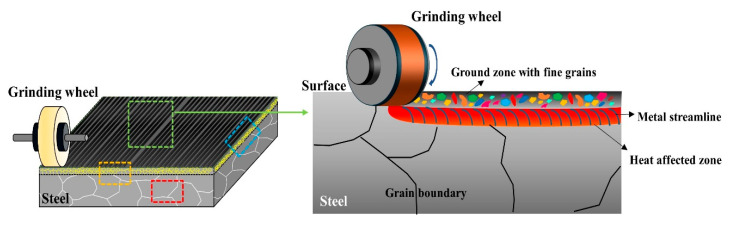
Schematic diagrams of the surface grinding on the steel slab and microstructural changes in the outer surface.

The material investigated in this study was high-Mn–low-Cr-based casting steel (POSCO technical research laboratories, Pohang, Republic of Korea), with a chemical composition of 0.3~0.4 wt.% C, 20~24 wt.% Mn, 0.2~0.3 wt.% Si, 2~3 wt.% Cr, 0.02~0.03 wt.% Ni, and balance Fe. After casting a square-shaped large-scale slab with dimensions of 700 mm in width, 4000 in length, and 225 mm in thickness, it was air-cooled to 200 °C. Subsequently, the surface of the cast slab was ground using a grinder (GRN-20, Shigiya, Schaumburg, IL, USA) with a ceramic bond white corundum grinding wheel (F46 of 300 mm in diameter and 25 mm in width). The grinding depth, feed rate, and grinding wheel speed were 0.1 mm, 20 mm/s, and 35,000 mm/s, respectively. Figure 1 depicts the schematic diagram of the grinding process on the steel slab.

### 2.2. Microstructure Characterization

The surface part of the ground steel slab was cut using electrical discharge machining to obtain samples (40 × 50 × 5 mm^3^) for microstructural examination employing FE-SEM (Hitachi, Tokyo, Japan), EBSD (Symmetry, Oxford, UK), and TEM (TECNAI 200, FEI, Amsterdam, The Netherlands). The cross-section morphology of the ground sample surface was examined in both the front view (grinding direction, GD) and side view (transverse direction, TD) of grinding, as indicated by the orange and blue dashed boxes in Figure 1. Additionally, the cross-section morphology below the damaged surface region, as indicated by the red dashed box in Figure 1, was also analyzed. Fine precipitates in the microstructure were characterized by the EBSD phase mapping and TEM diffraction pattern with a thin foil specimen. For EBSD analysis, the cross-section of the specimens was polished to 0.04 μm using a diamond suspension. The acceleration voltage, beam current, and step size were 20 kV, 1nA, and 40 nm, respectively.

### 2.3. Corrosion Testing and Analyses

To precisely comprehend the mechanism of corrosion-induced surface degradation in the samples, two types of corrosion tests were conducted: the periodic water spraying test and the simulated temperature–humidity exposure test.

In the periodic water spraying test, the surfaces of ground and unground samples were exposed to tap water (Ca: 19.7 mg/L, Mg: 4.5 mg/L, Na: 13.3 mg/L, K: 4.4 mg/L, Cl^−^: 13.3 mg/L, SO_4_^2−^: 21 mg/L) by spraying in a perpendicular direction once every 12 h for seven days. This causes a portion of the tap water to remain on the sample surface. The rationale for selecting tap water as the test solution is that, unlike distilled water, it effectively simulates the presence of Cl^−^, Na^+^, Ca, and other elements that may be present in the moisture condensing on the surfaces of steel produced in manufacturing plants located near coastal areas. After drying in air, the cross-sections of the samples were examined on days one, three, and seven of the testing periods. The corrosion product formed on the surface after seven days of testing was additionally characterized through X-ray diffraction (XRD, Bruker type diffractometer using a Cu anode) (New D8-Advance, Bruker, Ettlingen, Germany) with a thin film mode.

In a separately conducted simulated temperature–humidity exposure test, the conditions under which the specimens were exposed were categorized into four groups: summer–dry season (SD), summer–rainy season (SR), winter–dry season (WD), and winter–rainy season (WR). Summer and winter corresponded to the periods from June to July and December to January, respectively. The dry season was defined as the midpoint of a seven-day period without rain, while the rainy season was set as the midpoint of a five-day period with rainfall. Detailed variations in temperature and humidity within each group were based on data from the province of Jeonnam provided by the Meteorological Administration in South Korea, as shown in Supplementary Figure S1. These temperature–humidity exposures were conducted in a constant temperature and humidity chamber (TH-150U, Seoul, Republic of Korea). The number of corrosion-induced attacks per unit centimeter and the average depth of the attacks were determined by taking the average values from measurements at 10 different locations observed in the cross-sections of the corroded samples.

For a more detailed mechanistic understanding of the effect of fine precipitates (carbides) within the microstructure of the ground sample on corrosion behavior, additional annealing was conducted by heating to 1100 °C for 10 min using Gleeble simulator. This was followed by cooling with two different cooling rates (5 °C/s and 200 °C/s), resulting in two samples with different fractions of carbide precipitation. Typically, 200 °C/s corresponds to a sufficiently rapid cooling rate that can suppress the precipitation of carbides (e.g., when quenching the steel sample in water), while 5 °C/s represents a sufficiently slow cooling rate that allows for carbide precipitation (e.g., during natural cooling in air). Their corrosion behaviors were examined electrochemically using a potentiostat (Reference 600, Gamry, Warminster, PA, USA), and the corrosion parameters of corrosion current density (*i_corr_*) and polarization resistance (*R_p_*) were determined through the linear polarization resistance (LPR) test and electrochemical impedance spectroscopy (EIS) test, respectively. For these electrochemical measurements, a three-electrode system consisting of working (WE), counter (CE), and reference (RE) electrodes was employed. A Pt grid and a saturated calomel electrode (SCE) served as the CE and RE, respectively. Prior to the experiments, the samples serving as the WE were polished with a 2000-grit sand paper and ultrasonically cleaned in ethanol. The LPR test was performed by polarizing the samples from −20 to 20 mV vs. OCP at a scan rate of 0.2 mV/s in a 3.5% NaCl solution for 3 d, and potential–current density plots in semi-log format were obtained. The EIS test was conducted over the frequency range from 100 kHz to 10 mHz, applying an amplitude of ±10 mV AC potential vs. the OCP, and Nyquist plots were obtained. To ensure the reproducibility and repeatability of the electrochemical measurements, each type of test was repeated three times.

## 3. Results

### 3.1. Microstructural Examination of the Ground Sample

**Figure 2:**
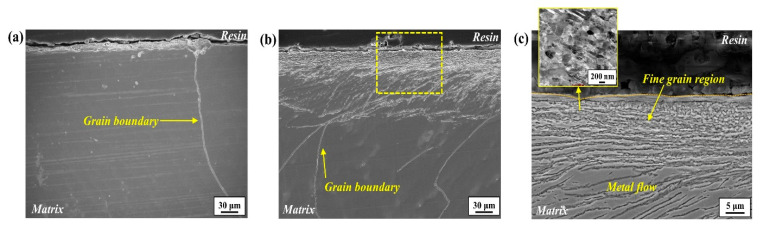
Cross-section morphologies in the side view of grinding for (**a**) unground and (**b**) ground samples ((**c**) magnified image of yellow dashed box shown in (**b**)).

Figure 2 presents the cross-section morphologies in the side view of grinding for (a) unground and (b,c) ground samples. In contrast to the unground sample, the ground sample exhibits a distinctive surface structure. It features a grinding-induced deformed region composed of a newly formed fine-grain area at the outermost layer, approximately 15~20 μm in thickness, with multiple metal streamlines (flows) skewed along the grinding direction, located just beneath it. Below the surface deformed region, a significantly coarse grain was observed, analogous to the unground sample, which is one of the characteristics of the large-sized casting slab.

**Figure 3:**
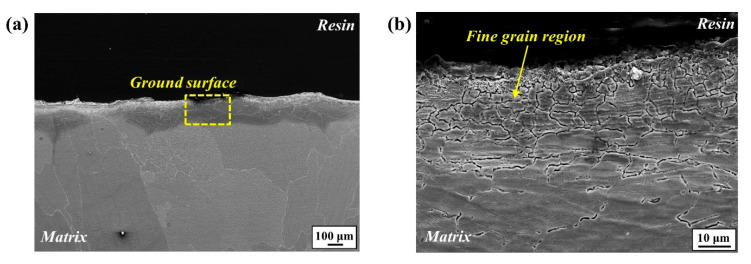
(**a**) Cross-section morphologies in the front view of grinding for ground samples ((**b**) magnified image of yellow box shown in (**a**)).

The newly formed fine-grain area was also observed in the cross-section image in the front view of grinding for the ground sample (Figure 3). In the low-magnification view (Figure 3a), the rough surface induced by the grinding process is also noticeable.

**Figure 4:**
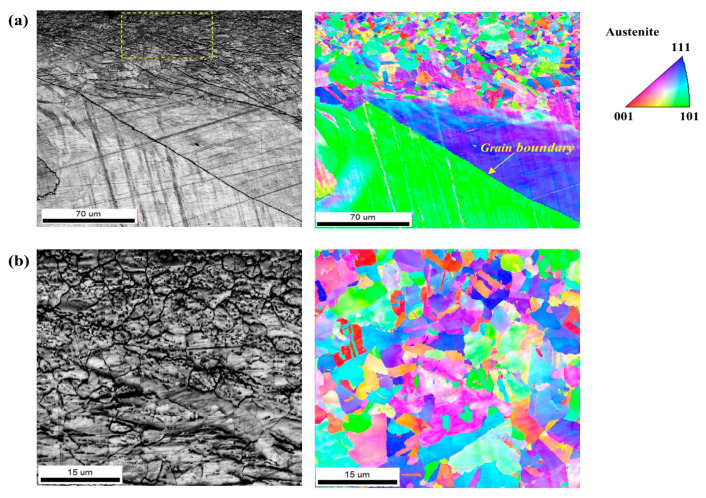
(**a**) EBSD grain-colored map of outer-surface part of the ground sample; (**b**) Magnified image of (**a**).

The EBSD grain-colored map, as shown in Figure 4, also indicates that randomly oriented fine grains with an average size of 5.5 μm were newly formed by the grinding process at the outermost surface of the casting sample with significantly coarse grains.

**Figure 5:**
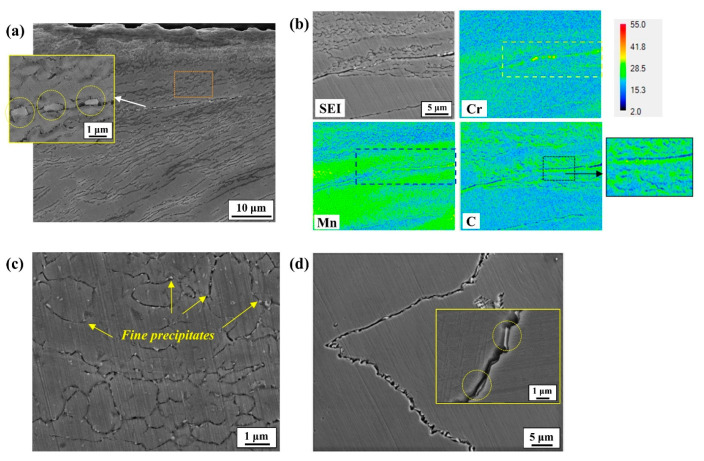
(**a**) Cross-section morphology in the front view of grinding for ground samples and (**b**) its EDS mapping; (**c**) magnified image of deformed region shown in (**a**); (**d**) magnified image of the undeformed region located below the deformed area shown in (**a**).

It is also noteworthy that another microstructural feature, aside from the surface deformed region, is the formation of fine precipitates enriched with Cr and Mn, mostly along the boundary areas, as observed from the cross-section in the front view of grinding (Figure 5). Although they were distributed throughout the matrix, their size varies: much finer ones were densely precipitated in the surface deformed region (Figure 5c) compared to the ones in the undeformed region, as observed in Figure 5d.

**Figure 6:**
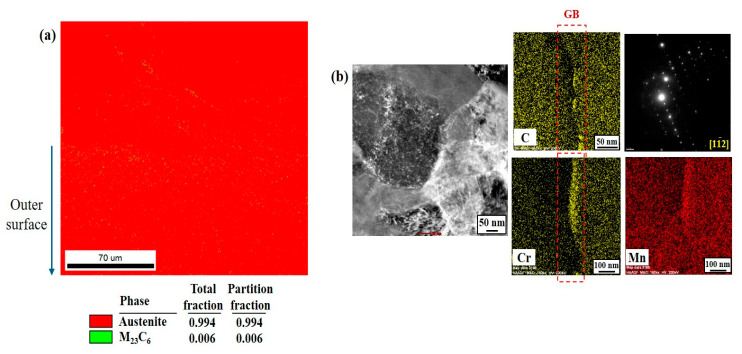
(**a**) EBSD phase map of deformed region located at the surface of ground sample, and (**b**) elemental distribution and diffraction pattern of fine precipitate, analyzed by TEM.

They were characterized by an EBSD phase map and TEM diffraction patterns, as presented in Figure 6a and 6b, respectively. EBSD analysis revealed that a smaller proportion of M_23_C_6_ particles, approximately less than 1%, were distributed throughout the austenite matrix. There was no apparent phase transformation induced by the grinding process. TEM analysis also indicates that the particles precipitated along the grain boundary correspond to Cr(Mn)_23_C_6_ with a cubic structure (lattice parameter (a,b,c): 10.65 Å). This implies that some Mn elements were partially substituted for the Cr element in the precipitates of the M_23_C_6_ type.

### 3.2. Corrosion Test: Periodic Water Spraying

**Figure 7:**
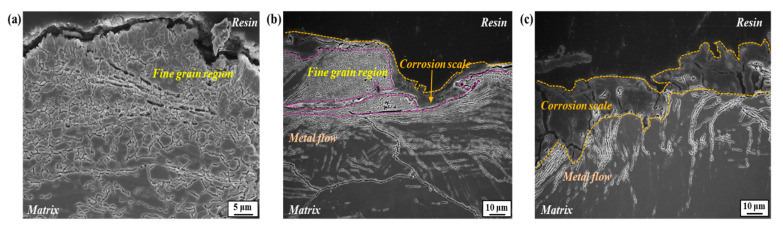
Cross-section morphologies after the periodic water spraying test for (**a**) one, (**b**) three, and (**c**) seven days.

Figure 7 presents cross-section morphologies showing corrosion-induced surface degradation with increasing testing time.

**Figure 8:**
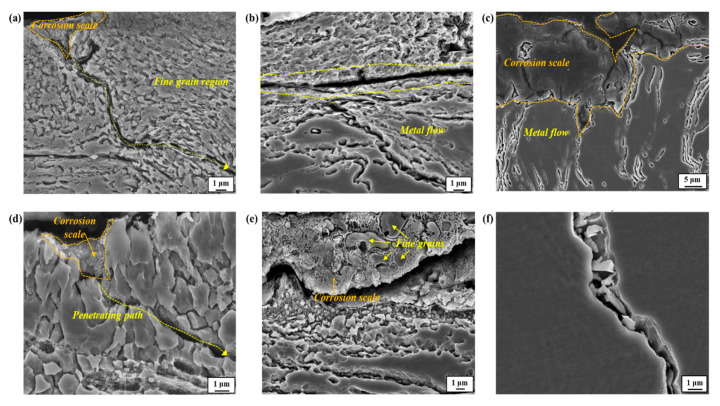
Magnified cross-section images after the periodic water spraying test for (**a**,**d**) one, (**b**,**e**) three, and (**c**,**f**) seven days.

The magnified images are also shown in Figure 8. In the early stage of one day (Figure 7 and Figure 8a,d), corrosion attacks propagated preferentially along the boundary of newly formed fine-grain areas, and the corrosion scale, characterized as Fe-based oxides/oxyhydroxides (refer to the XRD analysis presented in Supplementary Figure S2), remained in the propagation path. Over time, some portions of the fine-grain areas were detached, and the corrosion scale remained in those regions (Figure 7b). The magnified image shown in Figure 8e also indicates that the spaces between the detached fine grains are surrounded by corrosion scale. This suggests that corrosion-induced attacks in a penetrating form occurred primarily along the boundaries of fine grains. In the later stage of seven days (Figure 7c and Figure 8c), most of the fine-grain zones were detached by further corrosion processes, and additional corrosion damage occurred along the metal streamlines located beneath them. The upper surface was covered with a thick layer of corrosion scale. The magnified view shown in Figure 8f suggests that the primary propagation path of further corrosion attacks was the boundary where fine particles, characterized as Cr(Mn)_23_C_6_ (Figure 6b), were precipitated.

**Figure 9:**
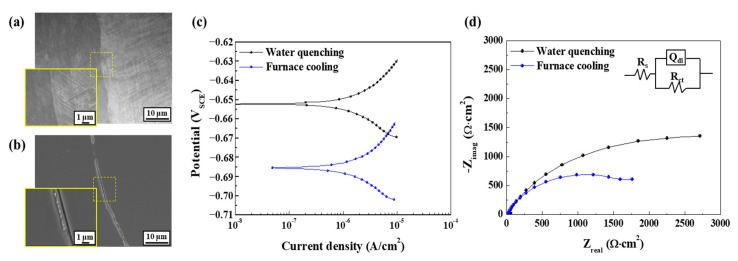
(**a**,**b**) Microstructure observations of the steel sample after annealing at 1000 °C for 1 h followed by (**a**) quenching and (**b**) furnace cooling; (**c**) linear polarization resistance curves and (**d**) EIS Nyquist plots for the two samples.

The contribution of particle density to aggravating the corrosion resistance can also be partially supported by the electrochemical test results, presented in Figure 9. In contrast to the annealed and quenched sample (WQ), where precipitation of particles (area fraction < 0.1%) was inhibited, the annealed and furnace-cooled sample (FC) with a higher density of particles (area fraction > 2%) exhibited a lower corrosion potential, higher *i_corr_*, and lower *R_p_*, as shown in Table 1. The polarization resistance and corrosion potential values exhibited meaningful variation, as the electrochemical test was repeated at least three times to ensure repeatability. The meaning of impedance parameters can be found in the previous study [12].

### 3.3. Corrosion Test: Temperature–Humidity Exposure

**Figure 10:**
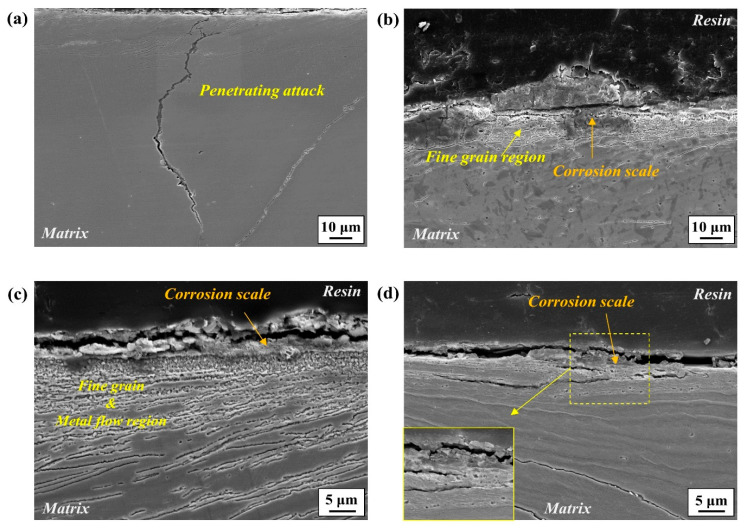
Cross-section images of the ground sample after simulated temperature–humidity exposure tests with (**a**) SD, (**b**) SR, (**c**) WD, and (**d**) WR conditions.

Figure 10 presents cross-section views showing the corrosion morphologies according to four environmental groups (SD, SR, WD, and WR) divided based on temperature–humidity combinations. The extent of corrosion damage appears to be less severe in winter conditions, and in the case of the WD sample, the surface deformed region composed of fine-grain zones and multiple metal streamlines remained nearly intact. On the other hand, under summer conditions, the severity of corrosion damage was significantly higher. Moreover, it is noteworthy that, compared to the SR sample with a high relative humidity of around 100%, the level of corrosion damage in the SD sample was much higher: the grinding-induced surface deformed zone was completely removed, and the corrosion damage in penetrating form along the boundary was noticeable. These corrosion behaviors were mechanistically quite similar to those in the periodic water spraying test. In the case of the SR sample, some portions of the surface deformed zone remained, and the corrosion damage did not develop into a penetrating form.

**Figure 11:**
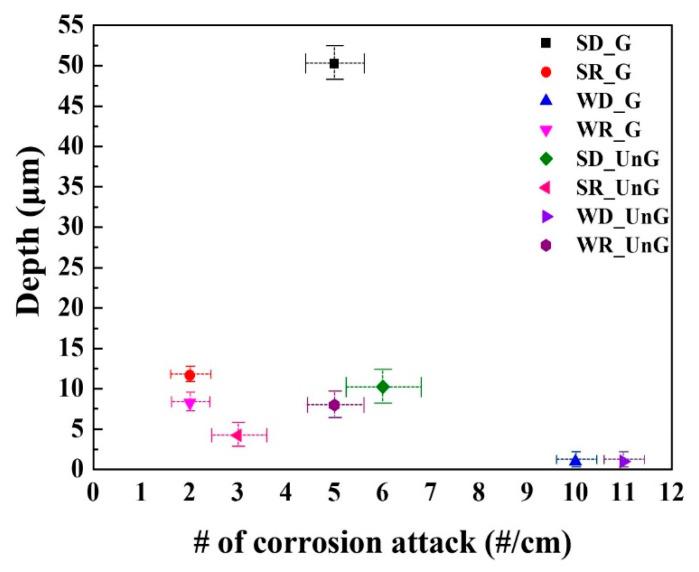
The density and average penetrating depth of corrosion damage after simulated temperature–humidity exposure tests (G and UnG denote the ground sample and unground sample, respectively).

Based on a number of morphological observations, the density (i.e., the number of corrosion attacks/cm) and average penetrating depth (μm) of corrosion damage were quantified, and their results are presented in Figure 11. In terms of the combination of density of corrosion damage and the penetrating depths, the SD condition indicates significantly higher severity compared to other conditions.

## 4. Discussion

The grinding heat and severe plastic deformation, generated by the abrasive grinding process for this high-Mn–low-Cr based cast steel resulted in the formation of a surface deformed region composed of a grain refinement layer and multiple metal streamlines. This grain refining can be interpreted as a result of dynamic recrystallization occurring under thermal–mechanical coupling conditions. The size of recrystallized grains is dependent upon the nucleation rate, which can be described as a function of strain rate and temperature, according to nucleation model proposed by Ding et al. [19]. This can be expressed as:

(1) $$\dot{n}\left(\dot{\varepsilon },T\right)=C{\dot{\varepsilon }}^{m}\exp\left(-\frac{Q_{act}}{RT}\right)$$

where $\dot{n}$, $\dot{\varepsilon },$ *C*, *m*, *Q_act_*, *R*, and *T* denote the nucleation rate, stain rate, rate constant [20], exponent, activation energy, gas constant, and temperature, respectively.

Hence, the fact that the recrystallized grains become much finer when the grinding process is conducted on the sample right after it reaches a higher temperature (600 °C) during the cooling process after casting can be understood (refer to the EBSD analysis in Supplementary Figure S3). It is considered that the fine-grain recrystallized layer contributes positively to other mechanical properties, such as strength, toughness, and fatigue resistance [21,22]. From an electrochemical perspective, however, fine grains with a high density of grain boundaries can have higher chemical reactivity and a higher local anodic dissolution rate than larger ones [23,24]. In light of this, it is expected that the early stages of corrosion occur preferentially along the boundaries of recrystallized fine grains, as noticed in Figure 7a and Figure 8a,d. However, the susceptibility of boundaries to corrosion may vary depending greatly on their physical and chemical characteristics. Among these characteristics, a major feature in this high-Mn–low-Cr cast steel alloy could be the precipitation of alloy carbides, mostly along the boundary areas. From a thermodynamic point of view, the addition of Cr to high-Mn steel favors the precipitation of M_23_C_6_ [11]. In the present cast alloy system, the alloy carbides were characterized as Cr(Mn)_23_C_6_ precipitated along the boundaries. According to Xu et al. [25], the M_23_C_6_ particle has a coherent interface and a cube–cube orientation relationship with the austenite grain from which it precipitated. This carbide can play a significant role in aggravating corrosion resistance from two aspects. Firstly, it serves as an electrochemical cathode when exposed to aqueous media, leading to higher anodic dissolution from the matrix due to microgalvanic action. Secondly, its precipitation causes the local depletion of Cr, resulting in corrosion propagation in a localized form, as observed clearly in Figure 8f. The annealed and FC sample with a higher density of the carbide exhibited a higher *i_corr_*, and lower *E_corr_* and *R_p_* can also be understood in this aspect. Specifically, the *E_corr_* values for the FC and WQ samples are −0.686 V_SCE_ and −0.652 V_SCE_, respectively, indicating a slightly lower value for the FC sample. Although the difference in *E_corr_* is minor, the *R_p_* values of 2550 Ω·cm^2^ and 3120 Ω·cm^2^ reveal a more noticeable contrast. The *i_corr_* values, which are inversely related to *R_p_* and commonly indicative of corrosion kinetics, are 8.18 μA cm^−2^ and 2.5 μA cm^−2^, with the FC sample exhibiting a value over three times higher. This suggests that the carbides (Cr(Mn)_23_C_6_) precipitated within the microstructure of the FC sample play a significant role in reducing corrosion resistance. This is further supported by the *R_ct_* values, which reflect the resistance to charge transfer reactions at the interface, derived from the EIS experiments (2150 Ω·cm^2^ for FC and 3820 Ω·cm^2^ for WQ), demonstrating an inverse relationship with *i_corr_*. Additional parameters obtained from the electrochemical tests can be found in Table 1 and Table 2. Although this electrochemical behavior is not directly indicative of the actual corrosion process, the fact that a higher precipitation of Cr(Mn)_23_C_6_ negatively affected the corrosion resistance of the steel sample can at least be accepted.

The metal streamlines formed beneath the recrystallized grains can also affect the corrosion behavior: they serve as a propagation path of corrosion attacks (Figure 7c and Figure 8c), due mainly to the high strain energy leading to a high density of dislocations. This can be supported in part by the higher kernel average misorientation (KAM) in the metal streamline areas, as shown in Supplementary Figure S3c. In general, highly deformed areas with high strain energy and metallurgical defects in metallic materials have higher exchange current densities for anodic and cathodic reactions by lowering the free energy [26,27,28], which is expressed as:

(2) $$i_{0}=Aexp\left(\frac{∆G^{*}}{RT}\right)$$

where $i_{0}$, *A*, and $∆G^{*}$ are exchange current density, Arrhenius pre-exponential factor, and energy barrier (activation energy), respectively.

**Figure 12:**
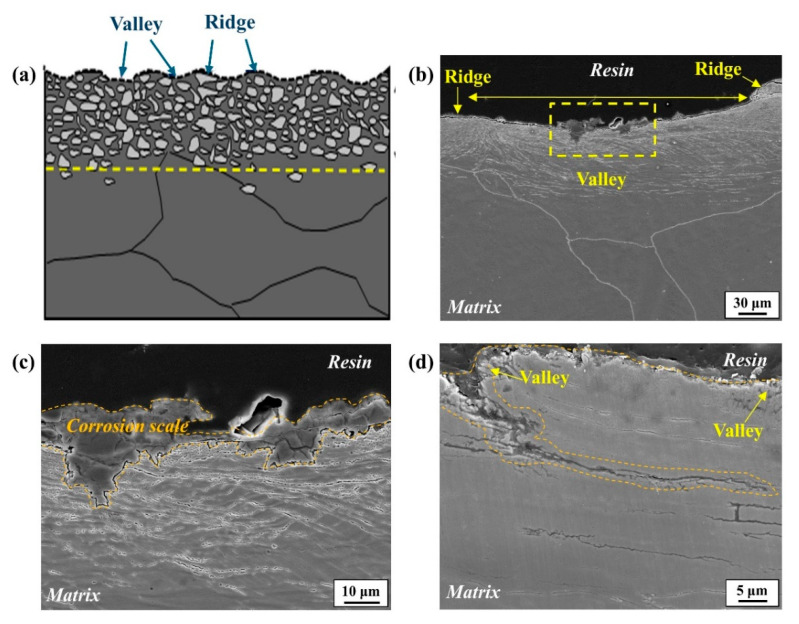
The cross-section morphologies of the ground sample, observed from the front view, showing the corrosion initiation and propagation behaviors: (**a**) schematic diagram of the cross-section of the outer surface; (**b**) corrosion initiation observed at the valley region; (**c**) magnified image of red dashed box shown in (**b**); (**d**) corrosion propagation.

Nevertheless, there may be uncertainty regarding why only localized areas are preferentially attacked in this alloy without forming a passive layer. For a precise understanding, the corrosion initiation site on the outer surface was observed, revealing that corrosion was initiated in the valley region where the surface was highly ground, as shown in Figure 12.

This valley could also be the region where a small amount of moisture can persist on the surface for longer times. Here, the hydrolysis reaction of Fe(Mn) can be facilitated, making it conducive to the reduction in local pH during the corrosion process [29,30], as described below:

M → M^n+^ + ne^−^ (M: Fe, Mn) (3)

H_2_O + O_2_ + e^−^ → 2OH^−^ (4)

Fe^2+^ + H_2_O → Fe(OH)_2_ + H^+^ (5)

Moreover, the formation of corrosion scale (Fe-based hydroxides) could be facilitated within a thin water film due to the extremely low solubility (solubility product ~ 10^−38^) [31]. Considering the higher volumetric expansion ratio of Fe-based oxyhydroxides in comparison with steel [32], the expansive force can create spaces on the steel surface, causing capillary action that facilitates the easy penetration of corrosive agents, including oxygen and moisture. Hence, the local propagating form of corrosion attacks with the rapid formation of corrosion scale in an initial stage can be understood. This was mechanistically represented in Supplementary Figure S4. To the best of our knowledge, this is the first experimental study to report the corrosion-assisted penetrating attack on ground high-Mn–low-Cr casting steel in humid environments, in contrast to the uniform corrosion behaviors typically exhibited by conventional low-alloyed steels.

Although this type of corrosion attack could occur more severely and frequently during the summer season, relative humidity is the most critical factor. It is worth noting that the highest severity occurs under relative humidity levels between 60% and 80%, rather than 100%. This can be interpreted in association with variations in the ease of oxygen reduction reactions (Equation (4)) on the steel surface depending on relative humidity. In contrast to conditions where relative humidity levels are around 100%, forming a bulk water layer on the steel surface, a relative humidity of approximately 60~70% allows a thin water film to form. Under these conditions, oxygen access to the steel surface becomes much easier [33], leading to more reduction reactions and overall corrosion attacks. This promotes the formation of corrosion products with a high volumetric expansion ratio, while capillary action further facilitates the penetration of corrosive agents, as discussed above. In spite of the formation of a thin water film under such humidity levels, the corrosion attacks can be limited under winter conditions of low temperature due to the lower diffusion kinetics of oxygen to the steel surface.

Based on the results, several technical strategies are recommended to mitigate corrosion-induced penetrating attacks on ground high-Mn–low-Cr casting steel slabs. These strategies include maintaining uniform surface roughness during the grinding process, controlling temperature and humidity (specifically, keeping relative humidity from 60% to 80% rather than 100%), and applying effective surface treatments. From this perspective, these findings offer valuable insights into the industrial applicability of high-Mn–low-Cr steels by significantly reducing surface damage (e.g., corrosion-induced penetrating attack).

## 5. Conclusions

This study investigated the metallurgical and environmental factors that cause corrosion-induced penetrating attacks on high-Mn–low-Cr casting steel subjected to surface grinding. Through various experimental and analytical tests, the underlying mechanism of this type of corrosion damage was interpreted. The main findings are summarized as follows.

The abrasive grinding process for this high-Mn–low-Cr based cast steel resulted in the formation of a surface deformed region composed of a grain refinement layer and multiple metal streamlines. Moreover, Cr(Mn)_23_C_6_ particles were densely precipitated at the boundary areas (recrystallized fine grain boundaries, metal streamlines, and coarse grain boundaries).

The early stages of corrosion occur preferentially along the boundaries of recrystallized fine grains, and the corrosion scale, characterized as Fe-based oxyhydroxides, was formed. With prolonged exposure to a corrosive environment, the fine-grain areas were gradually detached, and additional corrosion damage occurred along the metal streamlines located beneath them. The primary propagation path of further corrosion attacks was the boundary where Cr(Mn)_23_C_6_ particles were precipitated.

Macroscopically, the corrosion was initiated in the valley region where the surface was highly ground, facilitating the formation of corrosion scale (Fe-based oxyhydroxides) within a remaining thin water film. The expansive force caused by the higher volumetric expansion ratio of the corrosion scale detaches the surface deformed regions, enabling the easy penetration of corrosive media.

The local propagating form of corrosion attacks occurs more severely and frequently during the summer–dry (SD) season (i.e., relative humidity levels around 60% to 80%, rather than 100%) when a thin water film can form on the steel surface. The higher reduction rate based on oxygen accessibility to the surface during the SD season makes the steel highly susceptible to corrosion damage in humid environments.

It is recommended that technical strategies—including maintaining uniform surface roughness during the grinding process, controlling temperature and humidity (specifically, keeping relative humidity from 60% to 80% rather than 100%), and applying effective surface treatments—be implemented to mitigate corrosion-induced penetrating attacks on high-Mn–low-Cr casting steel slabs.

## Figures and Tables

**Table 1:** Mean values (*μ*) and their standard deviations (*σ*) of several fitted parameters obtained by curve-fitting to LPR data of the tested samples.

Sample	*i_corr_* (μA⸱cm^−2^)		*E_corr_* (V)		*R_p_* (Ω⸱cm^2^)		*β_a_* (V⸱decade^−1^)		*β_c_* (V⸱decade^−1^)	
	*μ*	*σ*	*μ*	*σ*	*μ*	*σ*	*μ*	*σ*	*μ*	*σ*
WQ	2.5	0.16	−0.6521	0.014	3.12 × 10^3^	19.26	0.035	0.003	0.035	0.004
FC	8.18	0.22	−0.6859	0.014	2.55 × 10^3^	23.24	0.12	0.002	0.08	0.002

**Table 2:** Mean values (*μ*) and their standard deviations (*σ*) of several fitted parameters obtained by curve fitting to Nyquist plots of the tested samples.

Sample	*R_s_* (Ω·cm^2^)		*Q_dl_* (×10^−4^ F·cm^−2^·s^n−1^)		*R_ct_* (Ω·cm^2^)		*n*	
	*μ*	*σ*	*μ*	*σ*	*μ*	*σ*	*μ*	*σ*
WQ	27.45	1.18	9.826 × 10^−4^	1.02 × 10^−5^	3820	23.2	0.7631	0.012
FC	20.24	0.84	1.123 × 10^−3^	9.75 × 10^−6^	2150	21.6	0.7343	0.018

## Data Availability

The data supporting the findings of this study are available from the corresponding author upon reasonable request.

## Supplementary Materials

The following supporting information can be downloaded at: https://www.mdpi.com/article/10.3390/ma17235922/s1, Figure S1: Variations in temperature and humidity of four environmental groups: (a) SD, (b) SR, (c) WD, and (d) WR. Figure S2: XRD pattern of ground sample, obtained after periodic water spraying test for 7 d. Figure S3: EBSD (a,b) grain-colored map and (c) KAM map of the sample ground at 600 °C. Figure S4: Schematic representation of corrosion-induced penetrating attack associated with corrosion scale.
