# Supplementary material for: Surface-Grinding-Induced Recrystallization and Metal Flow Causes Corrosion-Assisted Penetrating Attack of High-Mn–Low-CR Casting Steel in Humid Environments
Source: Materials (Basel). 2024 Dec 3;17(23):5922. doi: 10.3390/ma17235922 (PMC11643921; doi:10.3390/ma17235922)

Supplementary Materials

Surface grinding-induced recrystallization and metal flow causes corrosion-assisted penetrating attack of high Mn-low Cr casting steel in humid environments

Park et al.

*Corresponding author: E-mail: sjkim56@scnu.ac.kr

Figure S1. Variations in temperature and humidity of four environmental groups: (a) SD, (b) SR, (c) WD, and (d) WR.

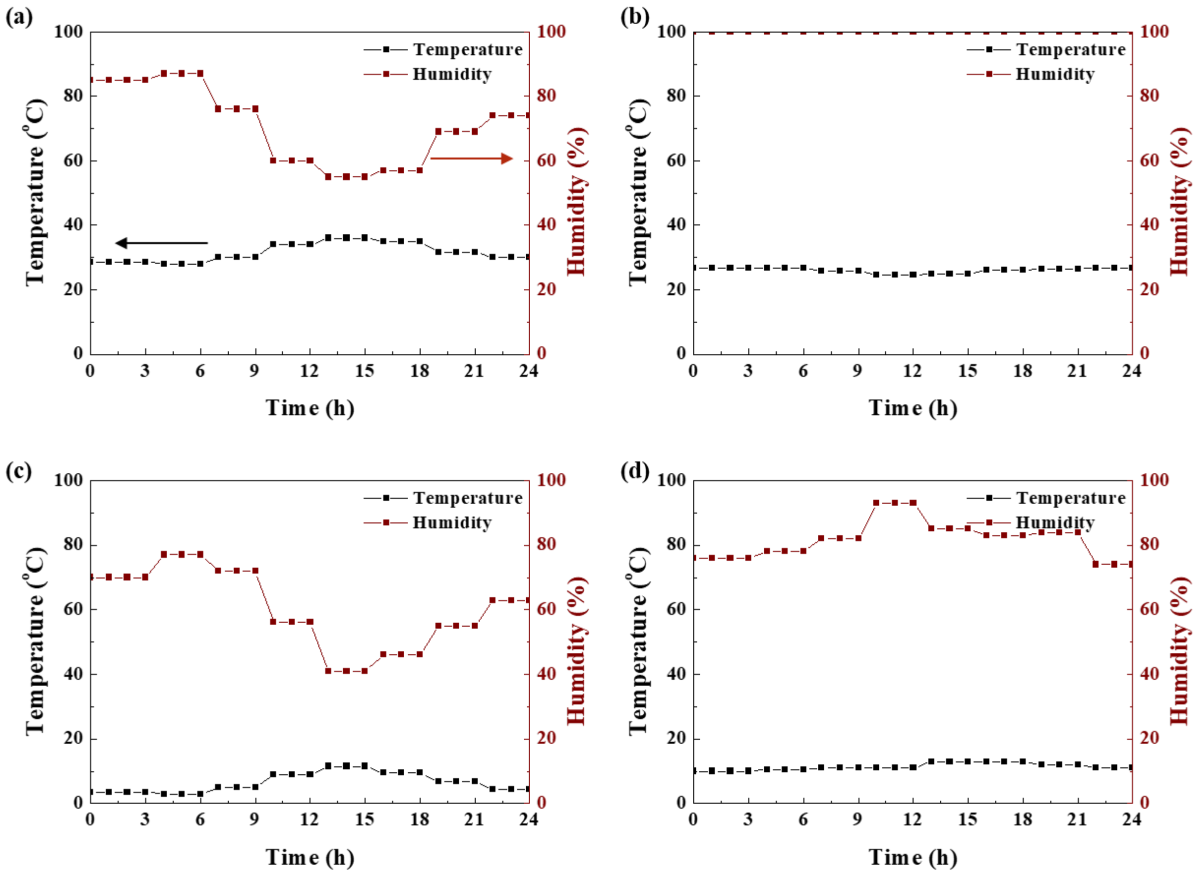

Figure S2. XRD pattern of ground sample, obtained after periodic water spraying test for 7 d.

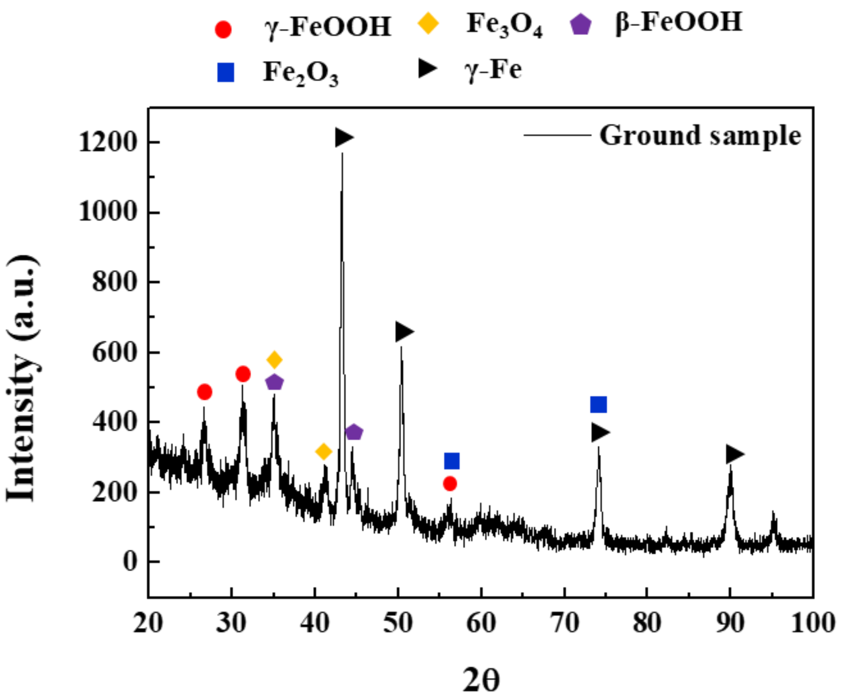

Figure S3. EBSD (a,b) grain-colored map and (c) KAM map of the sample ground at 600 ^{o}C.

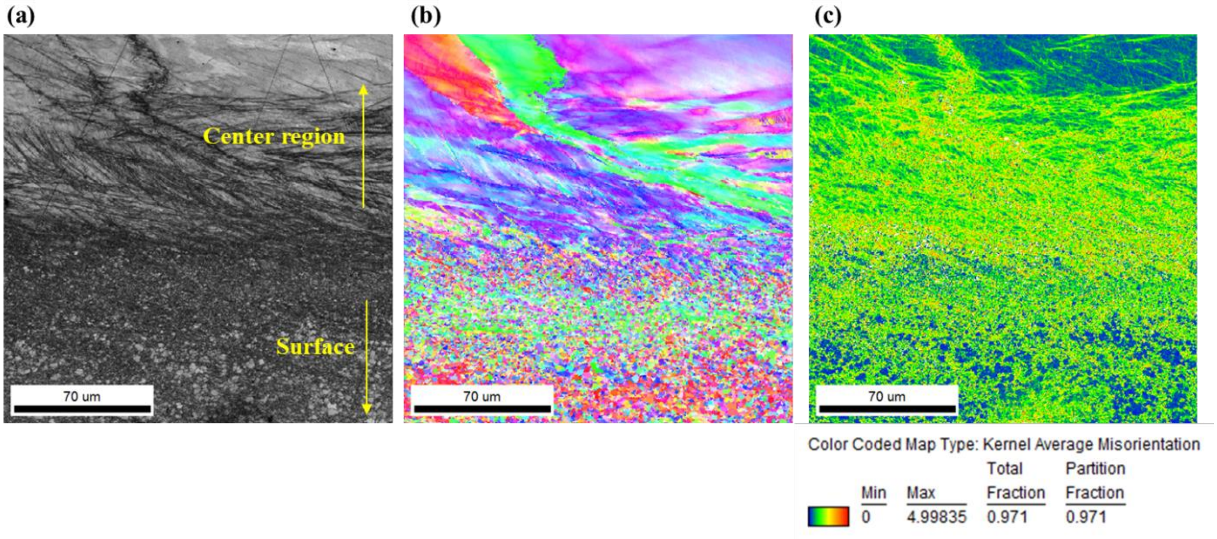

Figure S4. Schematic representation of corrosion-induced penetrating attack associated with corrosion scale.

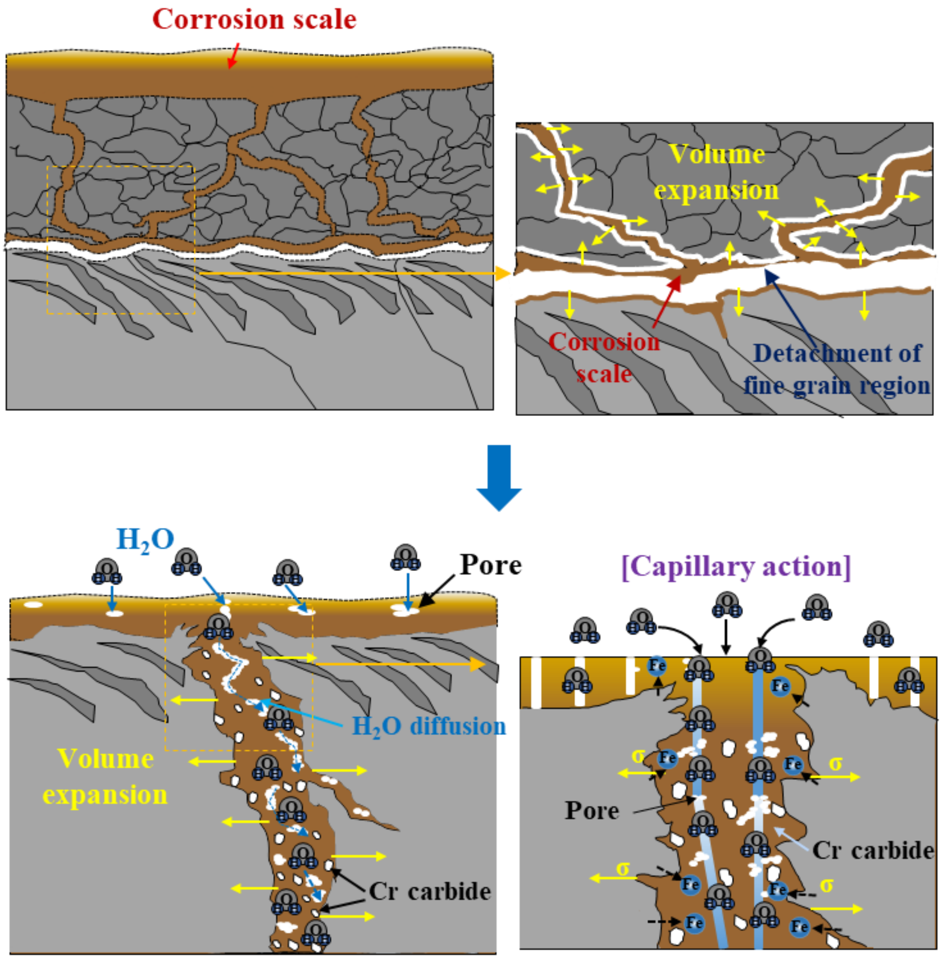

Supplement: Supplementary file 1 [file materials-17-05922-s001.zip › materials-3281226-supplementary.pdf]
